# Do epigenetic changes caused by commensal microbiota contribute to development of ocular disease? A review of evidence

**DOI:** 10.1186/s40246-020-00257-5

**Published:** 2020-03-13

**Authors:** Ashima Nayyar, Sofya Gindina, Arturo Barron, Yan Hu, John Danias

**Affiliations:** 1 Department of Cell Biology, State University of New York (SUNY) Downstate Medical Center, Brooklyn, NY USA; 2 Department of Ophthalmology, State University of New York (SUNY) Downstate Medical Center, Brooklyn, NY USA

## Abstract

There is evidence that genetic polymorphisms and environmentally induced epigenetic changes play an important role in modifying disease risk. The commensal microbiota has the ability to affect the cellular environment throughout the body without requiring direct contact; for example, through the generation of a pro-inflammatory state. In this review, we discuss evidence that dysbiosis in intestinal, pharyngeal, oral, and ocular microbiome can lead to epigenetic reprogramming and inflammation making the host more susceptible to ocular disease such as autoimmune uveitis, age-related macular degeneration, and open angle glaucoma. Several mechanisms of action have been proposed to explain how changes to commensal microbiota contribute to these diseases. This is an evolving field that has potentially significant implications in the management of these conditions especially from a public health perspective.

## Background

Many chronic non-infectious diseases like Alzheimer’s disease (AD), age-related macular degeneration (AMD), autoimmune uveitis (AU), and open angle glaucoma, have multifactorial etiologies that frequently include the interaction of environmental risk factors and genetic predisposition. Despite decades of linkage and family studies, only a limited number of polymorphisms or mutations have been identified which have a causative role in these conditions [40]. Even then, such genetic alterations account for only a small portion of disease prevalence [79].

In an effort to understand how the genetic background affects disease susceptibility and possibly determine environmental factors that contribute to such susceptibility, attention has been focused in recent years on the role of the human microbiome. The human microbiome project was established by the National Institutes of Health in order to identify the microbial composition in different body regions (e.g., oral, intestinal, nasopharyngeal, urogenital, and skin) [11, 69, 95]. Such attention is warranted as the microbiome accounts for 1–3% of human body weight and in aggregate comprises more than 100 trillion cells [113]. Furthermore, the microbiome is complex, dynamic, and potentially host specific [11]. The microbiome is comprised of diverse bacterial, viral, and eukaryotic species involved in host-microbe and microbe-microbe interactions [11, 113]. The commensal microbiome modulates nutrient acquisition, provides enzymes, adjusts immune system development, and serves as a protective barrier to foreign/opportunistic pathogens by competitive exclusion and production of antimicrobial substances [113].

Under physiologic conditions, commensal homeostasis is maintained via cross-regulation between the host and the resident microbiota [28, 61, 113]. Commensal microbiota constitution is determined by genetic inheritance and environmental factors (e.g., diet, smoking, antibiotic exposure, infection, and disease) [11, 65, 113]. For example, high fiber diets are linked to greater diversity of gut commensal microbiota, which limits the colonization by pathogenic bacteria that are associated with inflammatory bowel disease and colorectal cancer [13]. Similarly, the microbiome composition differs between babies delivered vaginally and via cesarean section; vaginal births allow for exposure to maternal vaginal and fecal bacteria causing subsequent predominance of Bifidobacterium species that is important for postnatal immune development [63].

Commensal bacteria help maintain a symbiotic relationship often with host cells via epigenetic modifications of host genes. For example, the intestinal microbiome can directly impact the intestinal epithelial cells (IECs), which line the large intestine lumen, through epigenetic changes in toll-like receptors (i.e., 5’CpG methylation) in order to prevent the triggering of an excessive inflammatory reaction [108]. IECs serve as both a physical barrier and frontline defense against pathogens by secreting antimicrobials and producing cytokines that regulate and recruit immune cells [108].

In recent years, the pathogenesis of diabetes mellitus, inflammatory bowel syndrome (IBS), atherosclerosis, obesity, liver disease, and cancer have been associated with a disbalance of commensal microbiota homeostasis [11, 113]. This homeostatic disbalance has been termed “dysbiosis.” Although originally the term dysbiosis was used to describe altered pathogenic bacteria in the gut, it is currently defined as “... qualitative and quantitative changes in microbial flora, their metabolic activity and their local distribution” [4, 44].

Dysbiosis can induce or exacerbate disease via toxic effects from direct invasion/infection or via epigenetic changes to host cells. At the cellular level, commensal microbiome-induced epigenetic changes commonly occur via either histone acetylation/deacetylation or DNA methylation [68].

Histone acetylation usually promotes active gene transcription while deacetylation represses gene expression. Bacteria can regulate histone acetyl modifications via production of short chain fatty acids (SCFAs) (such as acetic, butyric, and propionic acid), which are transported into the cell via monocarboxylate transporters. There they are metabolized and can function as substrates for acetylases or as inhibitors of histone deacetylases (HDAC) [15]. Acetate is known to promote acetylation of histone tail lysine residues [32] while butyrate can have an inhibitory effect on HDAC activity; both resulting in histone modifications and transcriptional regulation [89]. As an example of such epigenetic changes induced by bacteria, it has been shown that increasing dietary fiber is able to enhance gut colonization by butyrate-producing bacteria [30], which induce anti-inflammatory activity and beneficial effects in preventing obesity and insulin resistance by inhibiting intestinal macrophage HDAC activity [23].

SCFAs and other bacterial metabolic products (e.g., folate) can also cause DNA methylation. For example, SCFAs have been reported to correct aberrant expressions of adiponectin and resistin in high-fat diet-induced obesity by promoter methylation [71].

Epigenetic changes caused by bacterial metabolites may occur at sites remote from the site that the bacteria are actually present. For example, folate produced by Bifidobacterium species in the gut can enter the circulation and serve as a co-factor of DNA methyltransferases in many tissues around the body. Epigenetic changes, however, can also be caused by the microbiome indirectly through modulation of the immune or neural systems. SCFAs affect dendritic cells and macrophages for example, inducing IL-10 and retinoic acid which are known to have inhibitory actions on histone deacetylase activity [53] while they enhance T regulatory cell (Treg) expression leading to enhanced production of anti-inflammatory cytokines, modulation of antigen presenting cell (APC) function, and their ability to induce apoptosis in effector immune cells [72].

Similarly, commensal bacteria can cause secondary epigenetic changes by affecting neural activity. It is known that the gastrointestinal microbiota can influence cognitive function and behavior by direct reprogramming of the hypothalamus pituitary adrenal (HPA) axis [74]. Such changes in central nervous system (CNS) function may occur through gamma-aminobutyric acid (GABA), tryptophan, and monoamines such as serotonin, histamine, and dopamine produced by bacteria that can bind to receptors on neuronal and immune cells [12, 63, 74, 112].

The current review focuses on the eye for two reasons:

1. The eye is an immune privileged organ with little direct contact (except on its surface) with bacteria, fungi or viruses.
2. The eye is part of the CNS and thus understanding the underlying relationships and mechanisms in this organ can provide insights into the role of microbiome in other CNS pathologies.

We summarize evidence that links a number of important ocular pathologies with the microbiome, discuss possible mechanisms, and articulate some of the topics that need further exploration.

## Main text

### Anatomy of eye

**Fig. 1:**
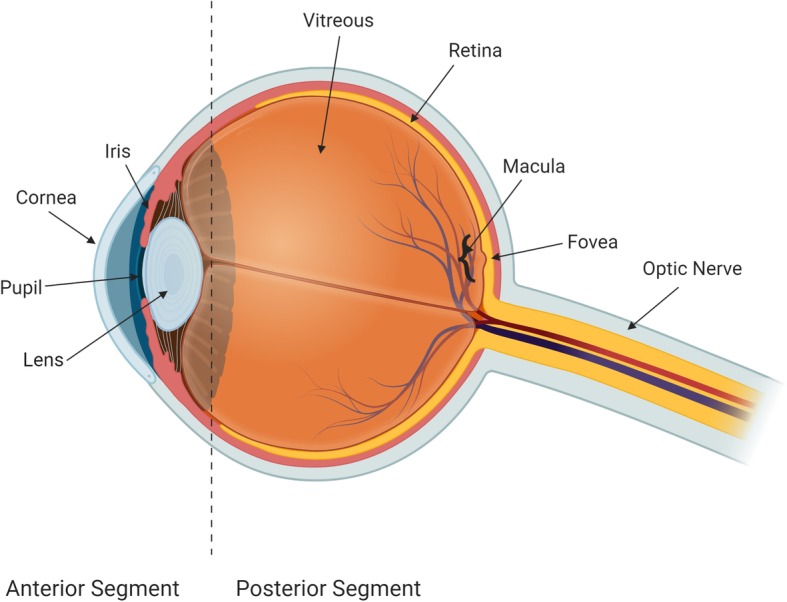
Anatomy of eye (adapted from National Eye Institute) [81]

Anatomically, the eye (Fig. 1) is comprised of the anterior segment (cornea, iris, ciliary body, and lens) and the posterior segment (vitreous humor, retina, choroid, sclera, and optic nerve). The anterior segment is responsible for focusing incoming light and for regulating intraocular pressure (IOP) via modulation of aqueous humor drainage. The posterior segment of the eye is responsible for visual perception and is comprised of a vascular layer (choroid) and the retina. The retina is composed of several neuronal cell layers and contains the photoreceptor cells responsible for light perception, with cones responsible for color perception and rods for black and white vision [39, 45]. Photons induce photoreceptor activation and hyperpolarization and this signal is transmitted to bipolar and horizontal cells followed by amacrine and ultimately ganglion cells [39, 45]. Retinal ganglion cell axons traveling within the optic nerve (cranial nerve II) relay this signal to the brain; thus the retina and optic nerve are part of the CNS.

As with the rest of CNS, the eye is immune privileged. The blood-retinal barrier (BRB) restricts free access of immune cells, microorganisms, and some molecules into the retina and is composed of the non-fenestrated capillaries of retinal circulation and the tight junctions between retinal pigment epithelial cells [98]. Similarly, the anterior segment is protected by the blood-aqueous barrier (BAB). Together, the BRB and BAB constitute the blood-ocular barrier. Vascular dysregulation or inflammation can lead to loss of such protection [73]. Blood supply to the eye is provided by branches of the internal carotid artery that progressively divide; the majority of vascular beds are highly anastomotic to ensure continued blood supply in the event of occlusion. The venous drainage of the eye occurs via vortex veins and the central retinal vein that merge with the superior and inferior ophthalmic veins to drain into the cavernous sinus [54].

The presence of lymphatics in the eye is controversial. Until recently, it was believed that there is no lymphatic outflow from the eye. However, recent research using lymphatic endothelium markers, combined with intracameral tracer injections, led to the discovery of lymphatic vessels in the corneal limbus and the ciliary body and choroid of the human eye [119]. The functional significance of these vessels under baseline conditions is unclear. However, in the context of this review, these lymphatic vessels may provide an alternative route for bacteria or their products to come in contact with CNS tissues. For example, signaling molecules secreted by both gut and oral microbiota have been found to be transferred via the lymphatic and systemic circulation where they can eventually affect behavior and modulate brain plasticity and cognitive function [27].

### Ocular microbiome and its role in microbial dysbiosis

Though the eye is an immune privileged site, an ocular microbiome does exist on its surface. Among healthy subjects, a broad spectrum of bacteria residing on the ocular surface have been identified. Twelve genera (e.g. *Pseudomonas*, *Corynebacterium*, *Acinetobacter*, *Staphylococci*, *Streptococcus*, *Streptophyta*, *Methylobacterium*, *Bradyrhizobium*, *Propionibacterium*, *Brevundimonas*, *Aquabacterium*, and *Sphyngomonas*) comprise 96% of the ocular microbiome [70]. However, recent studies have shown that more than 500 genera of bacteria are present on the conjunctiva [62]. The ocular surface microbiota can vary by ethnicity of host or become altered by environmental insults, and disease states [63, 78].

The innate immune system provides separation of the eye contents from the surface ocular microbiome via anatomic barriers, the complement cascade, and immune cells (i.e., macrophages and neutrophils). The tear film, which lubricates the ocular surface epithelia, contains antimicrobial compounds such as lysozyme, lactoferrin, immunoglobulin A (IgA), and lipocalin [76, 105]. Disruption of the ocular surface corneal and conjunctival epithelia can trigger ocular inflammation [16, 74]. A number of ophthalmic diseases are linked to pathogenic organisms. For example, corneal infiltrative events (CIE) can be caused by colonization of soft contact lenses with pathogenic bacteria, mainly Gram-negative species such as *Serratia marcescens* and *Haemophilus influenzae* [100]. Periocular bacteria can also enter the sterile intraocular compartments of the eye during surgery and cause diffuse infection and inflammation (i.e., post-operative infectious endophthalmitis) [10]. Operative conditions could also alter the ocular microbiota and heighten the risk for intraocular infection by pathogenic organisms [117]. However, by and large, bacteria do not reach any of the tissues inside the eye under normal conditions.

### Uveitis

Uveitis (inflammation of the uvea) is a condition that can acutely impair and endanger vision, thus requiring prompt treatment. It is often classified based on anatomic distribution (anterior, intermediate, and posterior), causation (infectious, non-infections, autoimmune, and drug induced), as well as chronicity (acute, recurrent, and chronic) [34, 104]. Uveitis has a prevalence rate of 5.4 per 1000 subjects in the USA and is associated with increased age and smoking history [38, 97]. Infectious causes of uveitis represent a minority of cases, while idiopathic/non-infectious/autoimmune uveitis, which represents the bulk of causes, is sometimes linked to systemic diseases [97].

The most common form of uveitis is acute anterior uveitis (AAU), accounting for 85% of cases. It is often associated with a leukocyte cell surface protein—human leukocyte antigen B27 (HLA-B27) [49, 95]. HLA cell surface proteins are responsible for presenting peptides to T-lymphocytes and thus regulating the immune response [95]. HLA variants are often associated with unique pathologies; for example, HLA-DQ2 is linked to the development of celiac disease. HLA-B27 is linked to a constellation of ailments, besides AAU, including psoriasis, ankylosing spondylitis, inflammatory bowel disease, Behcet’s disease, and reactive arthritis [5]. Thus, HLA heterogeneity across the general population serves as a marker for genetic predisposition for disease development. Recent evidence suggests that HLA variants are also associated with differences in the microbiome. For example, HLA-DQ2-positive infants have a different fecal microbiome when compared to infants that are HLA-DQ2 negative [97].

Most of the evidence hinting at a link between the microbiome and uveitis is based on decades-old clinical observations that certain diets exacerbate chronic uveitis. Recent analysis of clinical samples has provided evidence of a unique fecal metabolic phenotype in patients with AAU [49]. No significant quantitative differences in the species of gut microbiota between cases and controls were however detected, suggesting that metabolic differences reflect a change in microbiome function rather than its population profile [49].

To understand whether these associations are important in the pathogenesis of uveitis, animal models have been employed as they allow testing of specific hypotheses. An investigation comparing HLA-B27-positive transgenic rats and negative littermate controls showed significant differences in intestinal bacterial composition between transgenic and control animals, which was linked to pathology (with arthritis, spondylitis, rashes, and diarrhea) at several months of age [95]. Furthermore, the development of joint and intestinal disease manifestations was significantly reduced when animals were raised in a germ-free environment [95].

The exact mechanism by which an alteration in the gut microbiome can lead to AAU is unknown; however, there are several potential (non-exclusive) models worth exploring. For example, gut microbiome dysbiosis could cause increased intestinal permeability and a loss of immune homeostasis allowing the migration of bacterial byproducts or aberrantly activated immune cells to remote sites [95]. Microbiome dysbiosis can also induce a loss of local intestinal immune homeostasis leading to a lower activation threshold of immune cells and thus promoting a pro-inflammatory response. The eye may be affected via a molecular mimicry process, where a microbial antigen having homology with a self-antigen can induce an autoimmune response; thus, causing loss of tolerance toward ocular antigens that are normally sequestered behind the blood-ocular barrier [50]. Finally, disruption in barrier function, a known effect of bacterial cell wall components, can allow for the migration of microbial products and immune cells into the eye [50, 95].

To further understand how changes in the gut microbiome lead to uveitis development or progression, several groups have used inducible and spontaneous murine uveitis models (Phoebe [67, 96]). In the inducible experimental autoimmune uveoretinitis (EAU) model, mice are immunized with interphotoreceptor retinoid binding protein (IRBP) that is a uveitogenic antigen emulsified in complete Freund’s adjuvant [18, 25]. The intestinal permeability and microbiota composition were found to be altered during disease progression in EAU mice immunized with IRBP when compared to controls receiving adjuvant only [50]. Specifically, experimental animals had relative enrichment of *Prevotella*, *Lactobacilli*, and *Clostridium* species 2-week post-immunization, while control animals had relative enrichment of intestinal *Ruminococcus* and *Proteobacteria* species [50]. EAU mice immunized with IRBP showed associated changes in intestinal morphology and zonula-occludens-1 expression that correlated to increased intestinal permeability [50]. Raising the mice in a germ-free environment significantly reduced disease presentation and development [42]. Furthermore, oral, but not intraperitoneal, administration of broad-spectrum antibiotics (ampicillin, metronidazole, neomycin, and vancomycin) caused alteration of the intestinal microbiome and resulted in attenuation of uveitis (Phoebe [67, 80])*.* This broad-spectrum antibiotic combination is commonly used to reduce the intestinal bacterial load [26]. While ampicillin and metronidazole are well absorbed from the gut, and thus become systemically available, both neomycin and vancomycin are not absorbed from the intestinal tract and only affect the gut microbiome [26]. The use of antibiotics in this model was associated with an upregulation of regulatory T cells and a reduction in effector T cells and inflammatory cytokines [80]. The use of oral neomycin or ampicillin in isolation did not significantly reduce uveitis presentation nor upregulated regulatory T cells; metronidazole or vancomycin upregulated regulatory T cells, decreased inflammation, and uveitis severity [80]. These results therefore suggest a mechanism in which immune system modulation, specifically through T cells, can predispose to disease progression (P [66]; Phoebe [67, 80]). Antibiotics, affecting specific bacteria, alter the intestinal microbiome and thus reduce disease progression as described in this study.

In the R161H spontaneous uveitis mouse model, mice express a transgenic T cell receptor targeting IRBP (J [25]). This target antigen is sequestered in the immune privileged eye. Thus, for uveitis to occur, T cells must be exposed to IRBP and activated at an extraocular site [47]. Experiments using the R161H mice have revealed that antigens expressed by intestinal microbiota can lead to the activation of retina specific T cells in the gut lamina propria. T cells can then enter the eye to produce damage in a molecular mimicry process [47, 84]. Oral administration of broad-spectrum antibiotics (ampicillin, metronidazole, neomycin, and vancomycin) slowed the progression and attenuated the severity of uveitis and was associated with reduced Th-17 cell levels. This provides further evidence that gut microbiota can modulate the immune system causing T cell activation and a subsequent autoimmune response [47].

Although, it is difficult to discern the original trigger for uveitis, based on the data presented, it appears that disease progression may be linked to intestinal permeability and intestinal microbiome dysbiosis [50] through modulation of T cell activity ([91]; P [66]; Phoebe [67, 80]). Although findings in mouse models of uveitis may not necessarily translate to human disease, they provide an experimental model that can be used to explore relationships between uveitis and the microbiome [46, 114].

At a molecular level, recent studies have suggested that disease development in the EAU model is mediated through epigenetic changes. Tbx21 and Rorc are master transcription factors for differentiation of helper and regulatory T cells; therefore, dynamic changes in their expression would lead to alterations in the levels of these immune cells [33, 60]. Hypomethylation of these transcription factors was discovered in the retinas and RPE-choroidal tissues of EAU mice and was associated with a heightened production of Th1/Th17 specific cytokines (IFNγ and IL-17) [90]. These changes were correlated with a reduction in the expression of DNA methyltransferase (DNMT1) in these tissues [90]. Similarly, upregulation of miRNA-223 was detected in the EAU rat model [111]. miRNA-223 promotes inflammation through T cells and myeloid dendritic cells; furthermore, altered serum levels of miRNA-223 have been linked to microbiome dysbiosis [111]. In addition, comparisons of serum miRNA profiles between cases and controls detected a uveitis associated miRNA cluster [111]. This cluster of six miRNAs is linked to inflammatory signaling cascades, such as MAPK, FOXO, and VEGF [111].

Despite the studies cited above, our current understanding of the disease process remains incomplete. For example, it is unclear how primed T cells in the gut cause inflammation in the eye, which is normally immunologically privileged [114]. In addition, if the mechanism uncovered in mice is responsible for human disease, it is unknown what antigens trigger T cell sensitization in humans. Further investigation is required to determine whether specific microbiome-mediated epigenetic changes may trigger disease presentation in humans.

### Age-related macular degeneration

Age-related macular degeneration (AMD) is a progressive degenerative disorder leading to the loss of central vision as the disease preferentially affects the macular region of the retina that subserves central vision. Approximately 30–50 million individuals are affected with AMD globally with an estimated prevalence of 300 million by 2040 [93]. AMD is the most common form of maculopathy making it the leading cause of visual disability in the industrialized world and the third leading cause globally [55, 116]. The pathophysiology of AMD is not fully understood. It is considered to be multifactorial, involving genetic, environmental, and metabolic causes.

**Fig. 2:**
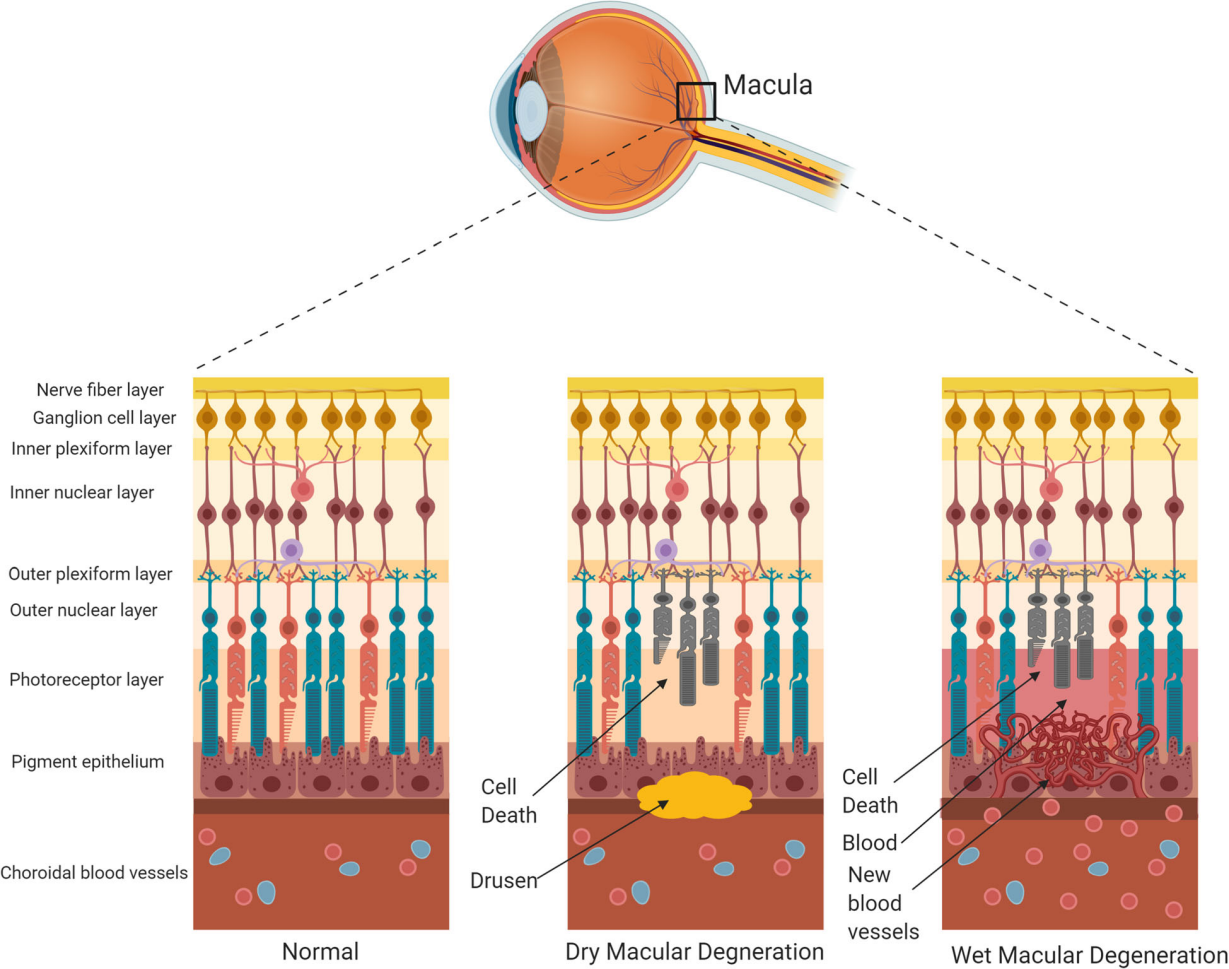
Schematic diagram of dry and wet forms of macular degeneration

The disease is categorized into dry or wet (neovascular) forms (Fig. 2). Dry AMD most often precedes the development of wet AMD [7]. In dry AMD, cellular debris called drusen accumulates beneath the retinal pigment epithelium (RPE) and Bruch’s membrane, which serves to separate the RPE from the fenestrated endothelium of the choriocapillaris [7, 106]. The RPE is comprised of non-dividing cells that transport nutrients and ions essential for photoreceptor cell maintenance [7, 106]. The presence of drusen eventually causes damage to the RPE leading to indirect photoreceptor cell death.

In wet AMD, there is choroidal neovascularization which can cause subretinal and/or intra-retinal fluid accumulation, hemorrhage, and lipid exudates all leading to RPE detachment and RPE and photoreceptor cell death [14, 107]. The exudative process can also directly damage photoreceptor cells [3, 7, 106].

The proliferation of aberrant vessels in wet AMD has been linked to an enhanced immune and vascular (via vascular endothelial growth factor stimulation) response [7]. The pathophysiology of dry AMD has been more difficult to discern, with several hypotheses proposed. These include an accumulation of lipofuscin (lipid-protein aggregates from incompletely degraded contents of phagolysosomes) at RPE cells promoting retinal oxidative damage [75, 92], mitochondrial defects causing intracellular and extracellular toxin accumulation similar to that of other neurodegenerative diseases [7], complement system dysregulation inducing inflammatory cell damage [92], and immune cell (microglia and macrophages) activation and infiltration [92]. Drusen components can induce nucleotide-binding domain, leucine-rich repeat-containing family, and pyrin domain-containing 3 (NLRP3) mediated inflammasome activation, causing the release of IL-1β and IL-18 in mouse retinal tissues [114].

Complement polymorphisms have been genetically linked to AMD [48, 114]. A variant of complement factor H (CFHY402H) is prevalent among patients with a predominantly dry form of AMD in the Caucasian population, whereas high temperature requirement factor A1 (HTRA1) is prevalent among those with a predominantly wet/neovascular form of AMD in the Asian population [48, 114]. Although the exact mechanisms through which complement polymorphic variants lead to AMD is still under investigation, it is believed that they allow for enhanced complement activation locally at the level of Bruch’s membrane, leading to endothelial cell damage and ischemia of the overlying RPE and retina [43].

In individuals not possessing high-risk complement polymorphic variants, it is hypothesized that AMD pathogenesis involves complement overactivation in response to stimuli at the retina and choriocapillaris. Potential triggers for the complement pathway include microbial colonizers. Bacteria (Bacillus *megaterium*) were discovered in some drusen of patients with AMD [114], though their origin has not yet been identified and these findings have not been confirmed by others. This pathogen is an aerobic firmicute ubiquitous to the environment. Intriguingly though, subretinal administration of the bacterium in non-human primates caused a drusen-like pathology to develop [114]. The discovery of bacteria at the site of pathology suggests that direct invasion of bacteria in the choriocapillaris is possible. Once in the local environment, these microbial organisms may release byproducts that induce epigenetic modifications and promote disease progression.

A link between pharyngeal microbiome dysbiosis and AMD development has also been reported [43]. Pathogens in the nasopharynx (e.g., Neisseria *meningitidis* and Streptococcus *pneumoniae*) are capable of enhancing the immune response to drusen deposition at Bruch’s membrane [43]. Although it is difficult to determine causality from these studies, the pharyngeal microbiome may play a role in AMD pathogenesis.

Known environmental risk factors associated with the development of AMD include aging, smoking, dietary intake, hypertension, and atherosclerosis. Diet is one of the modifiable risk factors for AMD. For example, following current dietary recommendations from the Age-Related Eye Disease Studies (AREDS and AREDS2) can reduce disease progression from dry to wet AMD for some patient groups [17]. As dietary intake of nutrients is affected by the intestinal microbiome, its composition in patients with AMD has been compared to that in controls without the disease. Patients with AMD have a relative enrichment in Oscillibacter, Anaerotruncus, *Ruminococcus torques*, and *Eubacterium ventriosum* species*,* while controls have an enrichment in *Bacteroides eggerthii* species [120]. It is interesting that Oscillibacter, Anaerotruncus, and Eubacterium species are associated with increased intestinal permeability, inflammatory changes during aging and elevated cytokine (IL-6 and IL-8) levels [120]. It was also reported that cases had elevated levels of *Streptococcus* and *Gemella* species along with reduced levels of *Prevotella* and *Leptotrichia* species, when compared to controls. These microbiome changes became more evident with increasing disease severity. There is speculation that intestinal microbial populations could potentially serve as a trigger for drusen formation or progression [120].

Gut microbiome dysbiosis is often linked to chronic inflammation and enhanced intestinal permeability, thus allowing bacterial products and pathogen-associated molecular patterns (PAMPS) to enter the circulation and interact with downstream pattern recognition receptors (PRRs), such as Toll-like receptors (TLR), to activate the innate immune response. Ocular cells, such as microglia, macrophages, and RPE cells, express PRRs and can thus become induced to an inflammatory state [120].

Alterations to the intestinal microbiome have also been shown to cause exacerbations of choroidal neovascularization in animal models. Experiments in mice were used to test the association between diet, obesity, microbiome alterations, and choroidal neovascularization. Mice fed either a regular diet or high-fat diet underwent laser-induced choroidal neovascularization as a model of AMD. Compared to regular diet fed controls, high-fat diet mice had a significant increase in weight associated with a 60% increase in the development of choroidal neovascularization [8]. An investigation into the microbiome composition of high-fat diet and regular diet fed mice revealed significant differences in Bacteroidetes/Firmicutes ratio with the former having a lower ratio than the latter. Furthermore, neomycin administration in the drinking water of high-fat fed mice resulted in changes to these gut microbial populations with a significant increase in the Bacteroidetes/Firmicutes ratio close to that of regular diet fed mice [8]. Despite high-fat diet mice still gaining weight, choroidal neovascularization was attenuated in animals receiving neomycin compared to those receiving vehicle [8]. In addition, oral neomycin administration was able to reduce the presence of sub-retinal mononuclear phagocytes (microglia and macrophages) in high-fat diet fed mice [8]. Therefore, changes induced by oral neomycin on intestinal bacterial flora could counteract the effects of a high fat diet on choroidal neovascularization [41].

Thus far, the microbiome has been linked to retinal changes at the tissue level that are potentially related to AMD pathogenesis. Research on a connection between the microbiome and intracellular modifications in AMD is currently lacking. Epigenetic events associated with AMD include post-translational modifications in the form of DNA methylation and histone acetylation in the retina [29, 37, 87]. Retinas from AMD patients show hypermethylation of the glutathione S-transferase P1 (GSTP1) promoter causing a reduction in mRNA expression of the two isoforms of GSTM (GSTM1 and GSTM5) [29, 37, 87]. GSTM proteins serve as a scavenger for reactive oxidative species and thus protect the retina from damage [29, 37, 87]. Thus, epigenetic repression via hypermethylation could lead to an increase in susceptibility to oxidative stress. Another epigenetic change seen in AMD is the hypomethylation of the interleukin 17 receptor C (IL17RC) promoter leading to increased expression. This receptor has been shown to promote pro-inflammatory cascades [29, 37, 87]. Finally, histone deacetylation has been shown to limit the accumulation of clusterin, a protein produced by the RPE and found as a major constituent in drusen [29, 87]. Though the environmental trigger for these epigenetic changes has not been defined, it is possible that the microbiome and its byproducts may influence such modifications.

### Open angle glaucoma

Glaucoma is a group of diseases characterized by progressive irreversible optic neuropathy, with retinal ganglion cell (RGC) axon and subsequent cell body degeneration, that results initially in peripheral visual field loss and can eventually lead to blindness [19]. More than 3 million Americans have glaucoma, of which 2.7 million aged 40 and above have open angle glaucoma (OAG), which is most prevalent in patients of African descent [109]. Glaucoma prevalence is increasing, with the number of patients having open angle glaucoma estimated to reach 65.5 million by 2020 [91]. The pathophysiology of most glaucoma forms involves an elevation in intraocular pressure (IOP) that is associated with a reduction in aqueous humor drainage in the anterior chamber of the eye. In open angle glaucoma, the anterior chamber angle (Fig. 1) is anatomically open but the outflow pathways are dysfunctional, causing IOP elevation. Chronic IOP elevation can lead to mechanical impairment, ischemia, oxidative stress, and inflammation within the optic nerve [109]. Extracellular matrix remodeling has also been shown at the lamina cribrosa and optic nerve head [2, 35, 94].

Other risk factors that contribute to OAG development include age, systemic diseases such as hypertension or hypotension, hyperlipidemia, diabetes, obstructive sleep apnea, thyroid disease, and genetic mutations ([77, 110]: [115, 118]). Mutations in the *MYOC*, *CYP1BI*, *FOXC1*, *PITX2*, *PAX6*, and *OPTN* genes have been associated with early onset glaucoma and appear to be causative although they have variable penetrance [20, 21].

A potential link between glaucomatous optic neuropathy and microorganisms has become a recent topic of investigation. A higher rate of gastric *Helicobacter pylori* infections was detected in glaucoma patients when compared to non-glaucomatous controls [22, 57]. HP infections had previously been detected in 88.2% of glaucoma patients [56]. Several mechanisms have been proposed to explain this correlation. *H*. *pylori* causes the release of various inflammatory factors, such as cytokines, ureases, and the neutrophil-activating protein VacA [83]. These, in turn, activate immune cells to produce cytokines, chemokines, proteolytic enzymes, nitric oxide, and reactive oxygen species. These effects cause further activation of microglia and differentiation into phagocytic macrophages at the optic nerve, ultimately damaging the ganglion cells [6, 58, 82]. Interestingly, it has also been reported that *H*. *pylori* eradication improves IOP control in addition to improved visual performance [58].

Other investigations into a possible relationship between microbiota and glaucoma detected a higher quantity of oral bacterial organisms (e.g., *Streptococci*) and worse oral health (fewer teeth) in patients with glaucoma compared to controls [9, 88]. In addition, in a study involving data from the Health Professionals Follow-up study, tooth loss within the 2 years prior to glaucoma diagnosis was associated with a 1.45-fold increased risk of POAG occurrence. The multivariate relative risk (MVRR) increased to 1.85 if tooth loss was accompanied by periodontal disease with bone loss during the same time period [85]. Thus, subclinical inflammatory processes caused by microbiome oral dysbiosis may trigger and potentially exacerbate glaucomatous damage based on clinical observations.

Periodontitis is a chronic inflammatory destruction of the gingival connective tissue attachment to the root surface, cementum, and adjacent alveolar bone, caused by complex polymicrobial dysbiotic subgingival biofilms that grow underneath the gums. Periodontitis has been linked to a number of other chronic human pathologies and conditions such as atherosclerosis [121], Alzheimer’s disease [52, 103], diabetes [99], rheumatoid arthritis [101], systemic lupus erythematosus [102], and obesity [99]. Reports of live periodontal bacteria detected in vascular endothelial cells suggest that commensal opportunistic pathogens may gain transient access to the vascular system during the course of the day [59, 64].

Periodontitis is also known to affect vascular reactivity and cause endothelial cell dysfunction, both of which have been suggested to play a role in glaucomatous neurodegeneration [1]. Activation of the local immune system within the retina and optic nerve head (ONH) in glaucoma could be the result of vascular changes that allow circulating immune components or bacterial components to gain access to these sites. Alternatively, periodontal bacteria-activated immune system components or circulating bacterial components could enter the ONH through the normal fenestrated capillaries and prime local microglia.

To test whether bacterial products could enhance glaucomatous neurodegeneration, low dose subcutaneous bacterial lipopolysaccharide (LPS) was administered in the hind foot pad in two separate glaucoma mouse models: the microbead induced-IOP elevation model and the spontaneous DBA/2J model [9]. This paradigm of LPS administration is more akin to chronic peripheral inflammation similar to that caused by dysbiosis of commensal microbiota [36]. LPS-activated Toll-like-receptor 4 (TLR4), which is associated with local inflammation and complement activation thus worsening glaucomatous degeneration (as measured by RGC and optic nerve axon counts); this was partially alleviated by Naloxone (TLR4 inhibitor) administration [9]. LPS administration was also associated with an increase in activated microglia in the optic nerve, which correlated with RGC loss [9]. Thus, bacterial products can lead to increased neurodegeneration of optic nerve axons via activation of local microglia [31].

Along with potential microglial involvement, T cells have also been implicated in glaucoma pathology. It has been shown that there is an elevation in anti-heat shock protein (HSP) autoantibodies such as HSP-27 antibody level and decreased antibody reactivity of α-enolase (a member of heat shock protein family) in glaucomatous human and animal tissues [51]. Furthermore, there is a significant elevation in HSP-27 and HSP-60 responsive T cells in human subjects with primary open angle glaucoma and normal tension glaucoma, compared to controls (H [24]). An inflammatory immune response can be induced at the retina by CD4^+^ T cell infiltration following their activation by commensal microflora [24]. Experiments suggest a chain of events in which transient IOP elevation induces infiltration of CD4^+^ T cells into the retina. These T cells, which target bacterial heat shock proteins (HSP’s), cross react with human HSP’s leading to the development of optic nerve neurodegeneration that can persist despite IOP normalization (H [24]).

At the molecular level, a number of epigenetic changes have been identified in association with glaucomatous optic nerve damage. These include abnormal histone acetylation/deacetylation in retinal ganglion cells (RGCs) that may be related to RGCs damage in glaucoma. For instance, histone deacetylase 2 and 3 expression were found to be significantly upregulated and histone H4 acetylase was found to be downregulated in RGCs [86]. This abnormal histone acetylation/deacetylation could potentially be caused either directly or indirectly as a result of commensal bacteria dysbiosis [16].

Thus, broadly speaking, inflammation caused by microbial dysbiosis can potentially lead to microglial activation within the retina and optic nerve via the following pathways: (1) direct (live) bacterial dissemination to the optic nerve and/or retina, (2) bacterial product dissemination to the optic nerve and/or retina, (3) effects secondary to changes in the vascular system, and (4) effects secondary to changes in the systemic immune system.

## Conclusions

The role of the microbiome in modulating human disease is slowly beginning to emerge. However, a number of critical barriers exist that hinder our ability to prove its role. These include:

1. Knowing *where* to look: Understanding which microbiome is important to individual pathologies. Data from association studies provide some initial clues, but often dysbiosis in a specific microbiome is associated with changes at other microbiome sites.
2. Knowing *when* to look: Understanding the temporal relationship between changes in a specific microbiome and development or exacerbation of disease. Cross-sectional studies are not ideal for detecting such temporal relationships. Many of the diseases in question develop over a period of months to years. The development of biomarkers that will measure long-term or past changes in the microbiome are sorely needed.
3. Knowing *what* to look for: Understanding whether specific bacteria/viruses/fungi are linked to pathology or whether a mere shift in the normal populations is more important. Association studies can provide clues for hypothesis generation but these need to be tested in well-controlled prospective studies.

Furthermore, disease heterogeneity, as well as, local and temporal variations of the microbiome often caused by environmental conditions (i.e. diet, exercise, humidity, etc.) compound the difficulty in establishing causal relationships.

Once an unequivocal relationship is established, understanding the mechanisms involved would become more straightforward although this task is by no means trivial. Use of animal models may be flawed because of different physiology. At the same time, in vitro studies often lack the complexity necessary to evaluate the interaction of multiple organ systems on the generation of pathology.

Finally, even when a causative link between the microbiome and an eye disease is confirmed, it will still take much work to develop rational interventions. Our knowledge of how to specifically affect microbial populations is limited at this time. Tools at our disposal (such as antibiotics or probiotics) are rather crude and often have significant side-effects. Improving our understanding of the interactions between individual members of the microbiome, and between them and the host will be critical in devising strategies to mitigate microbiome effects on disease development or progression. It is encouraging that research is rapidly expanding in all of the above areas but there is still a lot to be learned.

## Data Availability

Not applicable.
